# Supplementary material for: Sex Differences in B2 SINE RNA Expression and Their Role in Hippocampal Development
Source: Cells. 2026 Apr 30;15(9):816. doi: 10.3390/cells15090816 (PMC13162633; doi:10.3390/cells15090816)
Supplement: Supplementary file 1 [file cells-15-00816-s001.zip › cells-4153362-supplementary.pdf]

**Supplementary Data:**

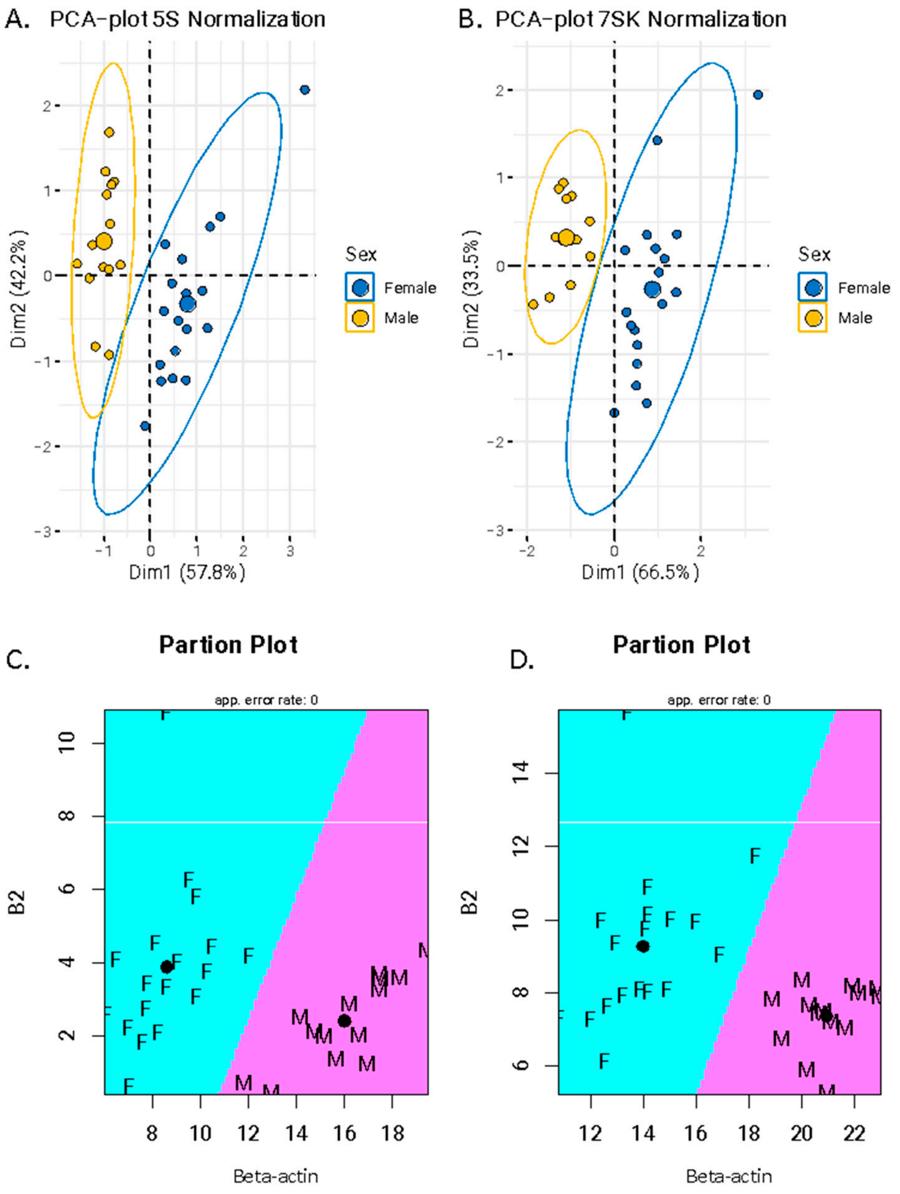


**Figure S1**. B2 SINE and beta-actin expression predictive models. A. Principal component analysis plot using expression values normalized to 5S and B. to 7SK PCA successfully segregates data into two clusters. C. Partition plot from linear discriminant analysis normalized to 5S and D. to 7SK. The models created from a subset of the data correctly predicts sex.

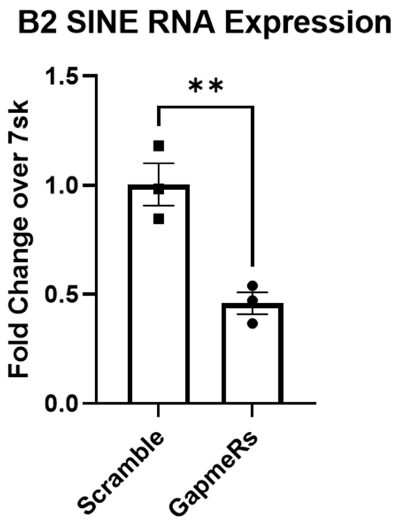


**Figure S2**. LNA GapmeR pool targets and knocks down B2 SINE RNA in primary hippocampal cells. Expression of B2 SINE RNA after either GapmeR pool or scrambled vector transfection. Data is represented as mean fold change over 7sk RNA ± SEM (n = 3/group) (**p<0.01).

In order to assess if the pooled B2 GapmeRs successfully knocked down B2 SINE RNA in primary hippocampal neurons after 6 hours, we transfected a subset of neurons for 6 hours, collected RNA, and tested for presence by RT-qPCR. We found a significant difference between the neurons transfected with the GapmeR pool and neurons transfected with a scrambled version. This shows that B2 SINE RNA is depleted after transfection with GapmeRs for 6 hours.

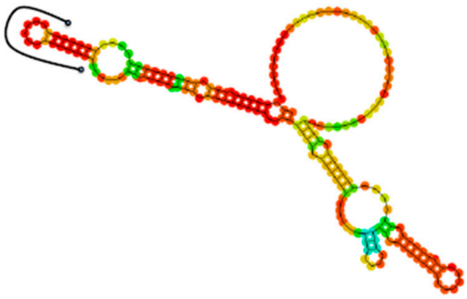


Androgen Response Element (ARE)

| Motif ID | Alt ID | Sequence Name | Strand | Start | End | p-value | q-value | Matched Sequence |
|---|---|---|---|---|---|---|---|---|
| 1 | AGAACANNNTGTTCT | B2_RNA | + | 31 | 45 | 0.00559 | 0.928 | GCACCCGACUGCUCU |
| 1 | AGAACANNNTGTTCT | B2_RNA | - | 31 | 45 | 0.00559 | 0.928 | AGAGCAGTCGGGTGC |

**Figure S3**. The secondary structure of B2 SINE RNA generated with RNAfold. B2 SINE RNA contains a putative ARE identified with motif analysis using FIMO from the MEME suite.

**Supplemental References:**


1. Jurka, J. *et al.* Repbase Update, a database of eukaryotic repetitive elements. *Cytogenet Genome Res* **110**, 462–467 (2005).

2. Bailey, T. L., Johnson, J., Grant, C. E. & Noble, W. S. The MEME Suite. *Nucleic Acids Res* **43**, W39–W49 (2015).

3. Gupta, S., Stamatoyannopoulos, J. A., Bailey, T. L. & Noble, W. S. Quantifying similarity between motifs. *Genome Biol* **8**, R24 (2007).

4. Grant, C. E., Bailey, T. L. & Noble, W. S. FIMO: scanning for occurrences of a given motif. *Bioinformatics* **27**, 1017–1018 (2011).


**Supplementary Methods:**

Primer sequences and GapmeR sequences are as follows. The sequences of B2 GapmeRs are 5′-UUC(dA)(dA)(dA)(dT)(dC)(dC)(dC)(dA)(dG)(dC)(dA)(dA)(dC)(dC)(dA)(dC)(dA)(dT)(dG)(dG)(dT)(dG)(dG)(dC)(dT)(dC)(dA)(dC)(dA)ACC-3′; 5′-AGU(dT)(dC)(dA)(dA)(dA)(dT)(dC)(dC)(dC)(dA)(dG)(dC)(dA)(dA)(dC)(dC)(dA)(dC)(dA)(dT)(dG)(dG)(dT)(dG)GCU-3′; 5′-GAG(dT)(dT)(dC)(dA)(dA)(dA)(dT)(dC)(dC)(dC)(dA)(dG)(dC)(dA)(dA)(dC)(dC)(dA)(dC)AUG-3; 5′-

AGC(dA)(dA)(dC)(dC)(dA)(dC)(dA)(dT)(dG)(dG)(dT)(dG)(dG)(dC)(dT)(dC)(dA)(dC)(dA)AC C-3′. The sequence of scrambled B2 GapmeR is 5'-CGGUGUGUGUAUCAUUCUCUAGUGU-3'. RN_7SK: Fwd 5'-TCGGTCAAGGGTATACGAGTAG-3' Rev 5'-TTTGGATGTGTCTGGAGTCTTG-3' RN_5S: Fwd 5'- CGTCTGATCTCGGAAGCTAAG-3' Rev 5'- CCTACAGCACCCGGTATTC-3'. B2 FWD 5′AGATGGCTCAGCGGTTAAGA-3'; B2 REV 5′-GACACACCAGAAGAGGGTATCA-3'

**MEME Suite:**

To reveal a potential putative binding site within the B2 SINE RNA consensus sequence, we used the MEME Suite. First, a *de novo* motif discovery was run using MEME with default settings, allowing 10 motifs on the B2 RepBase consensus sequence[1,2]. Generated *de novo* motifs were then compared against known eukaryotic vertebrate DNA motifs using TomTom[3]. Finally, a complementary FIMO search was used to investigate alignment with the AR consensus sequence within the B2 RNA sequence[4].
